# Supplementary figures and images for: Metabolic Potential, Ecology and Presence of Associated Bacteria Is Reflected in Genomic Diversity of Mucoromycotina
Source: Front Microbiol. 2021 Feb 15;12:636986. doi: 10.3389/fmicb.2021.636986 (PMC7928374; doi:10.3389/fmicb.2021.636986)

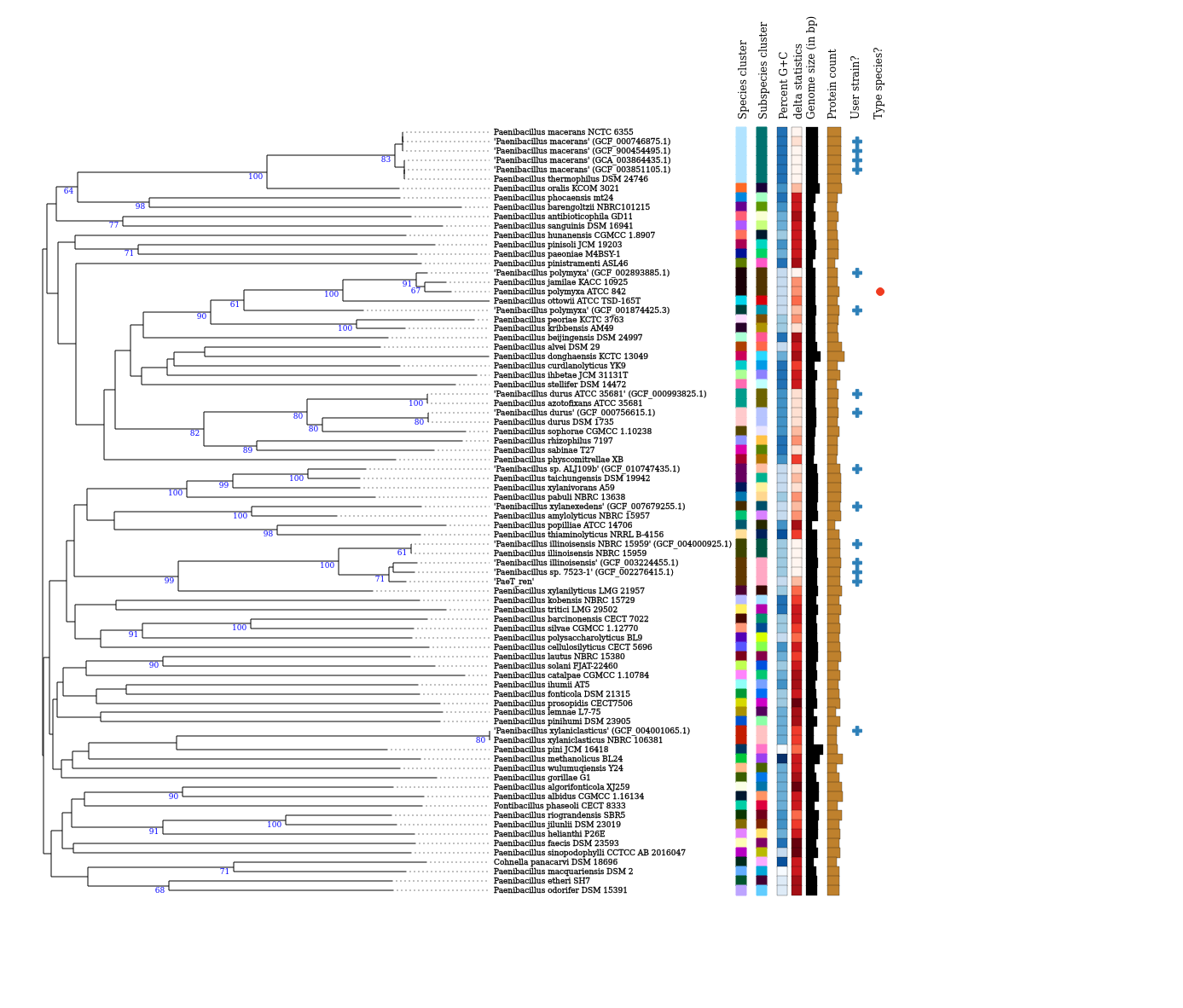

Supplement: Supplementary Figure 1 — Tree inferred with FastME 2.1.6.1 (Lefort et al., 2015) from Genome BLAST Distance Phylogeny approach distances calculated from genome sequences of Paenibacillus isolates (Meier-Kolthoff et al., 2013). The numbers above branches are pseudo-bootstrap support values > 60% from 100 replications, with average branch support of 47.8%. The tree was rooted at the midpoint by the program. Pae_T is the Paenibacillus sequenced as associated with Thamnidium elegans groups with P. illinoisensis strains. [file Image_1.PNG]
